# Effects of exercise training on gut hormone levels after a single bout of exercise in middle-aged Japanese women

**DOI:** 10.1186/2193-1801-2-83

**Published:** 2013-03-05

**Authors:** Shin-ya Ueda, Tadayoshi Miyamoto, Hidehiro Nakahara, Toshiaki Shishido, Tatsuya Usui, Yoshihiro Katsura, Takahiro Yoshikawa, Shigeo Fujimoto

**Affiliations:** 1 Department of Acupuncture, Morinomiya University of Medical Sciences, 1-26-16, Nankokita, Suminoe-ku, Osaka City, Osaka, 559-8611 Japan; 2 Laboratory of Research Promotion and Management, National Cerebral and Cardiovascular Center Research Institute, 5-7-1, Fujishirodai, Suita, Osaka, 565-8565 Japan; 3 Department of Elementary and Preschool Education, Osaka Seikei College, 3-10-62, Aikawa, Higashiyodogawa-ku, Osaka City, Osaka, 533-0007 Japan; 4 Department of Health and Physical Education, Kogakuin University, 2665-1, Nakano-machi, Hachioji City, Tokyo, 192-0015 Japan; 5 Department of Sports Medicine, Osaka City University Graduate School of Medicine, 1-4-3, Asahi-machi, Abeno-ku, Osaka City, Osaka, 545-8585 Japan

**Keywords:** Glucagon-like peptide-1, Peptide YY, Ghrelin, Exercise training

## Abstract

The purpose of this study was to investigate the effects of 12 weeks of exercise training on gut hormone levels after a single bout of exercise in middle-aged Japanese women. Twenty healthy middle-aged women were recruited for this study. Several measurements were performed pre and post exercise training, including: body weight and composition, peak oxygen consumption (peak VO_2_), energy intake after the single bout of exercise, and the release of gut hormones with fasting and after the single bout of exercise. Exercise training resulted in significant increases in acylated ghrelin fasting levels (from 126.6 ± 5.6 to 135.9 ± 5.4 pmol/l, *P* < 0.01), with no significant changes in GLP-1 (from 0.54 ± 0.04 to 0.55 ± 0.03 pmol/ml) and PYY (from 1.20 ± 0.07 to 1.23 ± 0.06 pmol/ml) fasting levels. GLP-1 levels post exercise training after the single bout of exercise were significantly higher than those pre exercise training (areas under the curve (AUC); from 238.4 ± 65.2 to 286.5 ± 51.2 pmol/ml x 120 min, *P* < 0.001). There was a tendency for higher AUC for the time courses of PYY post exercise training than for those pre exercise training (AUC; from 519.5 ± 135.5 to 551.4 ± 128.7 pmol/ml x 120 min, *P* = 0.06). Changes in (delta) GLP-1 AUC were significantly correlated with decreases in body weight (*r* = −0.743, *P* < 0.001), body mass index (*r* = −0.732, *P* < 0.001), percent body fat (*r* = −0.731, *P* < 0.001), and energy intake after a single bout exercise (*r* = −0.649, *P* < 0.01) and increases in peak VO_2_ (*r* = 0.558, *P* < 0.05). These results suggest that the ability of exercise training to create a negative energy balance relies not only directly on its impact on energy expenditure, but also indirectly on its potential to modulate energy intake.

## Introduction

The role of gut hormones in the treatment of obesity has been widely recognized (Derosa and Maffioli 2012; Field et al. 2009; Karra and Batterham 2010; Neary and Batterham 2009). Glucagon-like peptide-1 (GLP-1) is a satiety factor (Barrera et al. 2011; Gallwitz 2012; Torekov et al. 2011), which is released into the circulation after a meal in proportion to the amount of food consumed, and the major source of postprandial GLP-1 release is L-cells of the intestine (Barrera et al. 2011; Gallwitz 2012; Torekov et al. 2011). In addition, GLP-1 is the most powerful known incretin in humans, and manipulation of the GLP-1 system forms the basis of several major new treatments for type 2 diabetes (Barrera et al. 2011; Gallwitz 2012; Torekov et al. 2011). Peptide YY (PYY) is also recognized as a satiety factor, and is secreted from L-cells of the intestine after a meal and suppressed by fasting (Nguyen et al. 2011; Ueno et al. 2008). On the other hand, ghrelin is the only orexigenic endogenous hormone, and is secreted from the stomach due to fasting (Schellekens et al. 2012).

Interestingly, previous studies have revealed the inhibitory effects of acute exercise on the hunger associated with these hormones in healthy subjects (Broom et al. 2009; Cheng et al. 2009; Martins et al. 2007). We also demonstrated that a single bout of aerobic exercise caused significant increases in the plasma levels of GLP-1 and PYY, and decreases in subsequent energy intake in obese and non-obese subjects (Ueda et al. 2009a, b). Furthermore, increases in GLP-1 levels during exercise were significantly and negatively correlated with decreases in the amount of energy ingested (Ueda et al. 2009a). These findings suggest the intriguing possibility that exercise may partly function as a physiological regulator for hormone release or metabolism and thus lead to appetite control.

Previous training studies have examined changes in fasting hormone responses before and after an exercise training intervention, but not acute exercise (Jones et al. 2009; Kelly et al. 2009; Martins et al. 2010; Roth et al. 2005). To our knowledge, however, none have further examined if hormonal responses are altered after a single exercise bout. Clear understanding of the changes in gut hormones after a single bout of exercise by exercise training may potentially help us to develop new exercise programs for the prevention and treatment of obesity.

Therefore, the purpose of this study was to investigate the effects of 12 weeks of exercise training on gut hormone levels after a single bout of exercise in middle-aged Japanese women and to determine whether any changes correlated with the magnitude of exercise training-induced changes in body composition, fitness levels, and energy intake after the single bout of exercise.

## Methods

### Subjects

Twenty-eight healthy middle-aged women were recruited for this protocol. All subjects were lifelong non-smokers with a sedentary to moderately active lifestyle (less than one hour of intense exercise per day), and reported stable weight and lack of any special type of diet for the previous 6 months. None had any history of infectious disease for at least the 1-month period preceding the study, and none were taking medication. Subjects with a history of gastrointestinal, endocrine, cardiovascular, or psychological disease or type-1 or type-2 diabetes were excluded. Eight subjects were excluded due to trouble in home (n = 3), falls out of the training time (n = 4), and infectious disease (n = 1). Finally twenty subjects (mean ± SEM. 49.1 ± 0.8 years) were recruited for this study. Subject characteristics are shown in Table 1. All subjects provided written informed consent for participation in the study, which was approved by the Ethics Committee of Morinomiya University of Medical Sciences (Admitting No. 2012–002).

**Table 1:** **Subject characteristics**

	Pre	Post	Significance
Weight (kg)	68.0 ± 1.1	65.8 ± 1.0	***
Body mass index (kg/m^2^)	27.6 ± 0.4	26.8 ± 0.4	***
Body fat (%)	31.0 ± 0.8	28.6 ± 0.8	***
Peak VO_2_ (ml/kg/min)	23.5 ± 0.9	28.1 ± 0.8	***
Energy intake after the single bout of exercise (kcal)	835.6 ± 36.8	808.5 ± 41.8	NS (*P* = 0.08)

All values are described as mean ± SEM. ***P < 0.001: pre versus post exercise training.

### Study protocol

Several measurements were performed before and after 12 weeks of exercise training, including: body weight and composition, peak oxygen consumption (peak VO_2_), energy intake after the single bout of exercise, the release of gut hormones with fasting and after the single bout of exercise, glucose, insulin, and feelings of hunger and satiety.

### Exercise program

The aerobic exercise training was 80 min, three times per week for 12 weeks. Aerobic exercise was led by two trained fitness instructors and supervised by researchers. The training protocol consisted of 80 min with 10-min warm-up and flexibility exercise, 60-min aerobic exercise based on jogging (AR100, Minato Medical Science Inc., Tokyo, Japan) and cycling (Excalibur v2.0, Lode, Groningen, Netherlands), and a 10-min cool-down. The intensity of the aerobic exercise was approximately 65% of their maximal heart rate.

### Body composition

Body composition was measured by the extremities induction twelve-electrode bioelectrical impedance method (MC-190, Tanita Corporation, Tokyo, Japan).

### Peak VO_2_ measurement

Subjects performed a cycle ergometer (Excalibur v2.0, Lode, Groningen, Netherlands) ramp exercise test (20 W/min) to determine peak VO_2_ after 3 min rest on the ergometer and a 3 min 0 W warm-up as previously described (Ueda et al. 2009a, b). Peak VO_2_ was measured with an AE-310S Aeromonitor (Minato Medical Science Inc., Tokyo, Japan). Ventilatory and O_2_ consumption variables were calculated using the breath-by-breath method. The electrocardiogram and heart rate were continuously monitored using Dyna-Scope (DS-3140, Fukuda Densi, Tokyo, Japan) throughout the ramp exercise test. Perceived exertion was rated every minute with the Borg scale (Borg 1973). Attainment of peak VO_2_ was validated if two of the following four criteria were satisfied: (1) an oxygen uptake plateau despite increasing exercise intensity (≦150 ml / min); (2) a respiratory exchange ratio ≧1.10; (3) a maximal heart rate within 10 beats / min of the age-predicted maximal value; and (4) a Borg scale = of 10 (Borg 1973).

### Blood sampling and measures of appetite

**Figure 1:**
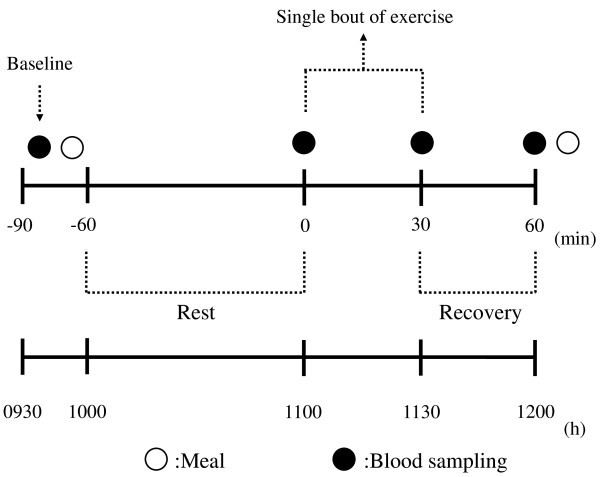
**Scheme of the experimental session.**

To control the subject's physical activity on the days prior to, and on the mornings of, the experiment, subjects were instructed to refrain from moderate to heavy exercise for at least 24 hours prior to each investigation. The design of the experimental session is shown diagrammatically in Figure 1. Subjects received a standard evening meal (instant noodles and a piece of cheese: 532 kcal, 13.9% protein, 26.6% fat, and 59.5% carbohydrate) at around 2100 h on the day preceding each study day. Subjects came to the laboratory at 0930 h and, after a 10 min rest period, a cannula was inserted into the antecubital vein and a fasting venous blood sample (baseline) was taken (20 ml). A standard breakfast (biscuits, yogurt, and jelly: 560 kcal, 16.5% protein, 19.3% fat, and 64.2% carbohydrates) was then served at 0950 h and participants remained seated quietly. At 1100 h (*t* = 0 min), the subject exercised on the cycle ergometer at 50% peak VO_2_ for 30 min. During these sessions and after the end of the exercise (*t* = 0, 30, 60 min), blood samples were collected. In addition, ratings of subjective feelings of hunger and satiety were reported on a 100 mm visual analogue scale (VAS) during the study period (*t* = baseline, 0, 30, 60 min) (Flint et al. 2000).

### Measurement of energy intake after a single bout of exercise

At 1200 h (*t* = 60 min), a test meal (instant pasta: 7.9% protein, 44.6% fat, and 47.5% carbohydrates (energy percent)) was provided and subjects were instructed to eat as much as they liked until satisfied, as described previously (Ueda et al. 2009a, b). In order to exclude the possibility that the amount of food eaten depended on its palatability, we asked all subjects which foods they liked prior to the study, and selected instant pasta as the test meal. We filled a small bowl with the test pasta and repeatedly filled the bowl with pasta before the participant had emptied it to ensure blindness to the amount of food eaten. No time limit was set for eating under either experimental condition. During the sessions, subjects and experimenters were instructed to abstain from talking about the meal. Participants were not overly informed that the true purpose of the present study was to assess feeding responses where possible until they had completed the protocol. After consumption of the test meal, any remaining food was weighed, and the amount determined was subtracted from the premeal value to obtain the total amount of food ingested. Absolute energy intakes from the test meal in both sessions (before and after the 12-week exercise training program) were then calculated from the amount of food eaten (1.15 kcal/g).

### Gut hormones, glucose, insulin, and hematocrit measurements

Blood samples were immediately transferred into disodium EDTA-treated tubes for measuring plasma glucose and hormones. In addition, aprotinin, a potent protease inhibitor, was added to samples at a concentration of 500 kIU/ml for the measurement of ghrelin. Test tubes were then centrifuged (KUBOTA 2010, KUBOTA Corporation, Tokyo, Japan) at 3000 r.p.m. for 15 min at 4°C immediately after collection, and plasma samples were stored at −80°C until used for hormone assays. Insulin was determined by the fully automated chemiluminescence method (CLIA). Glucose was measured using the enzymatic reference method with hexokinase. Plasma GLP-1 (GLP-1 (7–36) amide) and PYY levels were determined by EIA (Human GLP-1/PYY EIA kit, Yanaihara Institute Inc., Shizuoka, Japan). The ELISA for PYY quantified the total amount of both PYY_1–36_ and PYY_3–36_. Plasma acylated ghrelin levels were assessed by ELISA (Active Ghrelin ELISA kit, Mitsubishi Kagaku Iatron Inc., Tokyo, Japan). The interassay coefficients of variation for GLP-1, PYY, and ghrelin were each less than 18%. The sensitivities (minimum limits of detection) of GLP-1, PYY, and ghrelin were 0.062, 0.03, and 2.5 pmol/l, respectively. All sample measurements were performed in duplicate according to the manufacturers’ instructions. Hematocrit was measured using a Celltac alpha (Nihon Kohden Inc., Tokyo, Japan).

### Statistical analyses

All statistical analyses were performed using SPSS for Windows (SPSS Inc., Chicago, IL, USA). All data were normally distributed, assessed by the Kolmogorov-Smirnov test, and presented as means ± SE. Exercise training effects on each variable were tested by the Student’s paired *t*-test. In addition, to examine the effects of exercise training and time on the levels of hematocrit, glucose, insulin, gut hormones, and VAS scores, a two-way analysis of variance (ANOVA) with repeated measures was performed. If significance was detected, *post-hoc* multiple pair-wise comparisons (Tukey-Kramer test) were performed.

Areas under the curve (AUC) were calculated using the trapezoidal rule to assess total changes in each gut hormone, insulin, and glucose during each session (baseline to *t* = 60). The effects of exercise training on the AUC of each gut hormone, insulin, and glucose were assessed using the Student’s paired *t*-test. In addition, correlations between changes in the AUC of each gut hormone and other parameters were determined by simple correlation using Pearson’s correlation coefficient and stepwise multiple regression. Stepwise regression analysis was performed for other parameters as independent variables and the AUC of each gut hormone as a dependent variable. *P*-values less than 0.05 were considered significant.

## Results

Changes in anthropometry, body composition, fitness levels, and energy intake after the single bout of exercise are shown in Table 1. There was a significant reduction in body weight (*P* < 0.001), BMI (*P* < 0.001), and percentage body fat (*P* < 0.001) and a significant increase in peak VO_2_ (*P* < 0.001) after exercise training. Despite a tendency for lower energy intake after the single bout of exercise after exercise training, no significant changes were seen (*P* = 0.08).

### Fasting blood parameters

Changes in fasting glucose, insulin, and gut hormones are shown in Table 2. Exercise training resulted in a significant reduction in insulin (*P* < 0.001) and increase in ghrelin fasting levels (*P* < 0.01), but significant changes were not seen for glucose, GLP-1, and PYY fasting levels.

**Table 2:** **Fasting glucose, insulin, and gut hormones**

	Pre	Post	Significance
Glucose (mg/dl)	90.3 ± 2.5	89.6 ± 2.4	NS
Insulin (μU/ml)	11.8 ± 0.7	9.4 ± 0.5	***
GLP-1 (pmol/ml)	0.54 ± 0.04	0.55 ± 0.03	NS
PYY (pmol/ml)	1.20 ± 0.07	1.23 ± 0.06	NS
Ghrelin (pmol/l)	126.6 ± 5.6	135.9 ± 5.4	**

All values are described as mean ± SEM. ***P < 0.001, **P < 0.01: pre versus post exercise training.

### Blood parameters and feelings of hunger and satiety after the single bout of exercise

**Figure 2:**
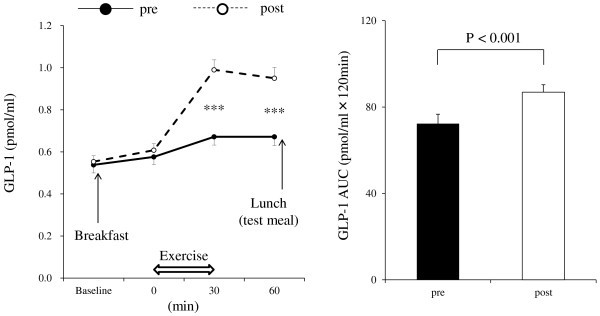
**Plasma level responses of GLP-1 to a single bout of exercise (*left*) and area under the curve values for GLP-1 (*right*).** Mean values ± SEM of each parameter are presented. Two-way ANOVA for repeated measures (*left*): Main effect of exercise training, *P* < 0.001; main effect of time, *P* < 0.001; interaction effect of exercise training × time, *P* < 0.001. ***P < 0.001, pre versus post exercise training.

**Figure 3:**
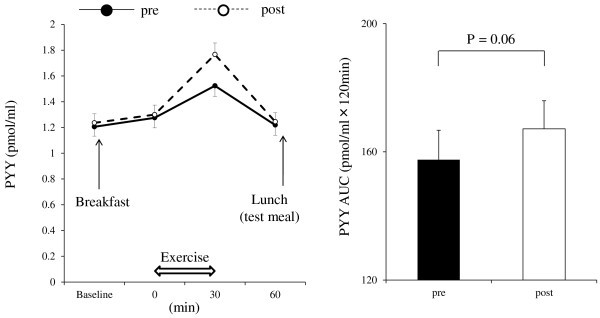
**Plasma level responses of PYY to a single bout of exercise (*left*) and area under the curve values for PYY (*right*).** Mean values ± SEM of each parameter are presented. Two-way ANOVA for repeated measures (*left*): Main effect of exercise training, *P* = 0.14; main effect of time, *P* < 0.001; interaction effect of exercise training × time, *P* = 0.40.

**Figure 4:**
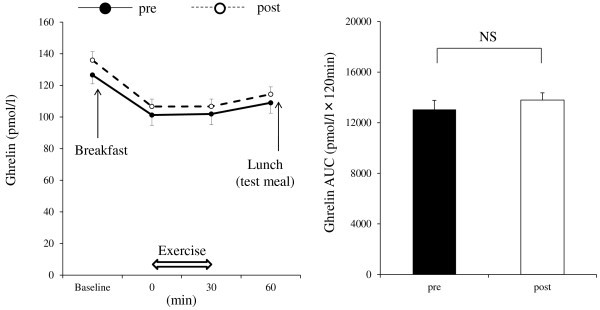
**Plasma level responses of ghrelin to a single bout of exercise (*left*) and area under the curve values for ghrelin (*right).*** Mean values ± SEM of each parameter are presented. Two-way ANOVA for repeated measures (*left*): Main effect of exercise training, *P* = 0.23; main effect of time, *P* < 0.001; interaction effect of exercise training × time, *P* = 0.97.

Significant changes in hematocrit were not observed over time during exercise. Hemoconcentration was thus unlikely to have occurred during the exercise sessions performed in the present study (data not shown). No significant differences in the levels of glucose and insulin were observed between pre and post exercise training throughout the course of observation (data not shown). Figure 2 shows the time courses of GLP-1 levels. A significant main effect of exercise training (*P* < 0.001), time (*P* < 0.001), and interaction (*P* < 0.001) were observed on GLP-1 levels. GLP-1 levels post exercise training after the single bout of exercise were significantly higher than those of pre exercise training (*post-hoc* test: *P* < 0.001, at *t* = 30 and 60 min). In addition, mean AUC values for GLP-1 post exercise training were significantly higher than those of pre exercise training (*P* < 0.001). Figure 3 shows the time courses of PYY levels. A significant main effect of time (*P* < 0.001), but no effect of exercise training or interaction, was observed on PYY levels. However, there was a tendency for higher mean AUC values for PYY post exercise training than those of pre exercise training (*P* = 0.06). Figure 4 shows the time courses of acylated ghrelin levels. A significant main effect of time (*P* < 0.001), but no effect of exercise training or interaction, was observed on active ghrelin levels. In addition, mean AUC values for active ghrelin did not change with exercise training. No significant differences in hunger, fullness, satiety, or motivation to eat were observed between pre and post exercise training throughout the course of observation (data not shown).

### Correlations of changes in gut hormone levels and each measurement parameter

Changes in (delta) GLP-1 AUC were significantly correlated with decreases in body weight (*r* = −0.743, *P* < 0.001), BMI (*r* = −0.732, *P* < 0.001), percent body fat (*r* = −0.731, *P* < 0.001), and energy intake after the single bout of exercise (*r* = −0.649, *P* < 0.01) and increases in peak VO_2_ (*r* = 0.558, *P* < 0.05) (Table 3). In contrast, no significant correlations between the delta of AUC values for PYY and ghrelin levels and changes in each measurement parameter were observed (data not shown). To investigate which variables accounted for these associations with AUC of each gut hormone, stepwise multiple regression analysis was performed. Consequently, body weight (r^2^ = 0.552, *P* < 0.01), BMI (r^2^ = 0.536, *P* < 0.01), and percent body fat (r^2^ = 0.535, *P* < 0.01) were selected as predictive variables of AUC values for GLP-1 levels.

**Table 3:** **Correlations between incremental GLP-1 responses and changes in each measurement parameter**

	r	***P***
⊿ Weight (kg)	−0.743	< 0.001
⊿ Body mass index (kg/m^2^)	−0.732	< 0.001
⊿ Body fat (%)	−0.731	< 0.001
⊿ Peak VO_2_ (ml/kg/min)	0.558	< 0.05
⊿ Energy intake after	−0.649	< 0.01
the single bout of exercise (kcal)		
⊿ Fasting glucose (mg/dl)	−0.214	NS
⊿ Glucose AUC (mg/dl)	−0.229	NS
⊿ Fasting insulin (μU/ml)	−0.337	NS
⊿ Insulin AUC (μU/ml)	−0.301	NS

## Discussion

The objective of the present study was to investigate the effects of 12 weeks of exercise training on gut hormone levels after a single bout of exercise in middle-aged Japanese women and to determine whether any changes correlated with the magnitude of exercise training-induced changes in body composition, fitness levels, and energy intake after the single bout of exercise. The following findings were obtained: 1) Exercise training resulted in a significant increase in acylated ghrelin fasting levels, with no significant changes in GLP-1 or PYY fasting levels. 2) A significant increase in GLP-1 levels and a tendency toward an increase in PYY levels after the single bout of exercise were observed after exercise training. 3) Changes in (delta) GLP-1 AUC were significantly correlated with decreases in body weight, BMI, percent body fat, and energy intake after the single bout of exercise and increases in peak VO_2_.

Recent studies have begun to shed light on the previously unknown roles of a single bout of exercise in energy intake or appetite regulation through gut hormone release (Broom et al. 2009; Cheng et al. 2009; Martins et al. 2007; Ueda et al. 2009a, b). For the development of new programs for the prevention and treatment of obesity, it is more important to examine whether exercise training also has an impact on the circulating levels of gut hormones and energy intake control as well as a single bout of exercise. In one study, a 12-week supervised exercise training program (five times per week, 75% maximal heart rate) resulted in a significant reduction in body weight and increase in fasting acylated ghrelin levels, with no significant changes in fasting GLP-1 and PYY levels (Martins et al. 2010). Our findings suggest that exercise training resulted in a significant increase in acylated ghrelin fasting levels and no significant changes in GLP-1 and PYY fasting levels in middle-aged Japanese women, consistent with data from a previous study (Table 2). On the other hand, it has been reported that an 8-month supervised aerobic training program decreased percent body fat and raised fasting PYY levels (Jones et al. 2009). Roth et al. (2005) observed a significant increase in fasting PYY levels in obese children who were successful in losing weight after a 1-year diet and exercise training. In addition, changes in fasting ghrelin levels by exercise training were not consistent across these studies (Foster-Schubert et al. 2005; Jones et al. 2009; Kelishadi et al. 2008; Kim et al. 2008; Konopko-Zubrzycka et al. 2009; Leidy et al. 2004; Mackelvie et al. 2007; Martins et al. 2010; Mizia-stec et al. 2008; Morpurgo et al. 2003; Santosa et al. 2007). However, most studies showed a compensatory increase in fasting ghrelin levels in response to reductions in body weight (Foster-Schubert et al. 2005; Kelishadi et al. 2008; Kim et al. 2008; Konopko-Zubrzycka et al. 2009; Leidy et al. 2004; Martins et al. 2010; Mizia-stec et al. 2008; Santosa et al. 2007). For example, whereas total ghrelin fasting levels were not affected by aerobic exercise training for 5 days without reductions in body weight (Mackelvie et al. 2007), fasting plasma levels gradually increased during 12 weeks of aerobic and resistance exercise with significant decreases in body weight and fat; and such reductions were strongly associated with increased fasting ghrelin levels (Kim et al. 2008; Leidy et al. 2004). For longer term exercise training where weight reductions were achieved over one year without caloric restriction, fasting ghrelin levels increased with weight loss, again suggesting a role for ghrelin in the adaptive response constraining weight loss (Foster-Schubert et al. 2005). Such discrepancies may stem from the fact that exercise training-induced changes in fasting levels of total ghrelin, acylated ghrelin, and PYY depended not only on the duration or intensity of the exercise but also on dietary conditions.

In two elegant studies by Donnelly et al. (2003) and Potteiger et al. (2003), 16-month of supervised exercise training by young women did not lead to changes in body weight. However, our findings suggest that a 12-week exercise training (three times per week) resulted in a significant decrease in body weight and percent body fat in middle-aged Japanese women (Table 1). Although we cannot provide a definite explanation for such a difference, inconsistent results were due to ethnic differences. Previous studies that have examined the association between the tryptophan-to-arginine (Trp64Arg) variant of the β3-adrenergic receptor gene and the magnitude of weight loss achieved by exercise intervention have yielded inconsistent results (Fumeron et al. 1996; Kogure et al. 1998; Rawson et al. 2002; Sakane et al. 1997; Shiwaku et al. 2003; Tchernof et al. 2000; Yoshida et al. 1995). All of the positive results have been reported from Japan (Kogure et al. 1998; Sakane et al. 1997; Shiwaku et al. 2003; Yoshida et al. 1995); and null results were reported from France (Fumeron et al. 1996) and the United States (Rawson et al. 2002; Tchernof et al. 2000), suggesting that the inconsistent results were due to ethnic differences. However, to the best of our knowledge, there are no studies dealing with this topic; thus our hypothesis remains speculative.

Martins et al. (2010) observed a tendency for higher postprandial GLP-1 and PYY levels in overweight/obese men and women after 12 weeks of exercise training. Kelly et al. (2009) reported that an increased PYY response to glucose ingestion was shown in older obese insulin-resistant adults after 12 weeks of exercise training and Chanoine et al. (2008) observed that an acute GLP-1 response to a liquid meal was shown to be enhanced by a 5-day aerobic exercise training program in normal weight and overweight adolescents. Similarly, our present findings first demonstrated that a significant increase in GLP-1 levels and a tendency toward an increase in PYY levels after the single bout of exercise were observed after 12 weeks of exercise training (Figures 2, 3). These results are likely to be important for developing new exercise programs for the prevention and treatment of obesity. However, because the physiological mechanisms underlying these changes in response to a stimulus have not yet been fully understood, further investigation is warranted. Additionally, we also should have included an experiment without acute exercise to elucidate the pure effect of exercise training on the gut hormone response to the breakfast intake. This could provide a better understanding of the interactions between energy intake, acute exercise, and exercise training on the secretion of gut hormones.

Another important outcome of this study was the fact that increases in AUC values for GLP-1 levels were significantly correlated with decreases in body weight, BMI, percent body fat, and energy intake after the single bout exercise and increases in peak VO_2_ (Table 3). Previously, we reported that reductions in energy intake after the single bout of exercise were significantly and negatively associated with total increases in GLP-1 concentrations during the single bout of exercise (Ueda et al. 2009a). In this study setting, significant increases in GLP-1 levels after the single bout of exercise were preceded by 12 weeks of exercise training. In other words, these results suggested that the ability of exercise training to create a negative energy balance relies not only directly on its impact on energy expenditure, but also indirectly on its potential to modulate energy intake.

There were some potential limitations to the present study. First, we did not calculate the energy expenditure during whole time course from breakfast until lunch time in each session, but simply expressed as amount of energy ingested at lunch. In the present study, we focused on how much energy subjects consumed at lunch after a single bout of exercise. If we can calculate relative energy intake, results can be used to further understand the interactions between energy balance and exercise training on the secretion of gut hormones. In a future study, we intend to calculate relative energy intake Second, we designed the present study, including the timing of energy intake and time of the single bout of exercise, based on our previous paper (Ueda et al. 2009a). Therefore, because the rather unusual eating pattern with only 2 hours between substantial meals may have had effects on the results of this study, it cannot be applied directly to general meal patterns. Further studies are required on the timing of energy intake and the single bout of exercise on gut hormones such as GLP-1, PYY, and ghrelin. Additionally, we cannot rule out the possibility that cognitive or environmental factors affected our findings, although we attempted to carefully exclude such confounding variables by the choice of study design. When allowed to eat *ad libitum*, obese subjects consumed more food items than subjects with a normal weight (Wing et al. 1978). In fact, in previous studies of appetite in the obese, subjects were instructed to eat *ad libitum* (Martins et al. 2007, 2010). However, under these circumstances, food intake can be biased by cognitive factors such as the belief that ‘food is a reward for exercise’ (King 1999). In the present study, a common test meal of noodles was therefore prepared so that subjects would be unaware of food intake during the test (Ueda et al. 2009a, b). In addition, prior to the study, we confirmed that the test meal was palatable to all participants. Third, in the present study, to evaluate gut hormones and energy intake after a single bout of exercise, subjects performed ergometer cycling before and after the exercise training. The intensity of the single bout of exercise was then adjusted to 50% peak VO_2_ (same relative intensity). Therefore, although mean% peak VO_2_ and heart rate during the single bout of exercise was not different between pre and post exercise training, mean workload post exercise training during the single bout of exercise was significantly higher than that of pre exercise training (*P* < 0.001, from 43.5 ± 0.9 to 48.1 ± 0.8 W). Additionally, to identify a true exercise training effect, it will be necessary to match the workload between pre and post exercise training sessions.

In conclusion, our findings showed that, in middle-aged Japanese women, GLP-1 and PYY levels after the single bout of exercise were increased by exercise training. In addition, increases in GLP-1 levels were significantly correlated with decreases in body weight, BMI, percent body fat, and energy intake after the single exercise bout and increases in peak VO_2_. These results suggested that the ability of exercise training to create a negative energy balance relies not only directly on its impact on energy expenditure, but also indirectly on its potential to modulate energy intake. A better understanding of the role of exercise training in energy intake regulation may lead to a more effective prescription of exercise training for weight control.
